# A Novel Digestive Proteinase Lipase Member H-A in *Bombyx mori* Contributes to Digestive Juice Antiviral Activity against *B. mori* Nucleopolyhedrovirus

**DOI:** 10.3390/insects11030154

**Published:** 2020-03-01

**Authors:** Shang-Zhi Zhang, Lin-Bao Zhu, Ling-Ling You, Jie Wang, Hui-Hua Cao, Ying-Xue Liu, Shahzad Toufeeq, Yu-Ling Wang, Xue Kong, Jia-Ping Xu

**Affiliations:** 1 School of Life Sciences, Anhui Agricultural University, Hefei 230036, China; 18755148780@163.com (S.-Z.Z.); zhulinbao@163.com (L.-B.Z.); 13063306714@163.com (L.-L.Y.); wangjie_3001@163.com (J.W.); chh18856960204@163.com (H.-H.C.); Liuyingxue8@163.com (Y.-X.L.); toufeeq@163.com (S.T.); 15755072270@163.com (Y.-L.W.); kx18895706038@163.com (X.K.); 2 Anhui International Joint Research and Developmental Center of Sericulture Resources Utilization, Hefei 230036, China

**Keywords:** *Bombyx mori*, BmLipase member H-A, *Bombyx mori* nucleopolyhedrovirus, antivirus

## Abstract

Previous studies have revealed that some proteins in *Bombyx mori* larvae digestive juice show antiviral activity. Here, based on the label-free proteomics data, BmLipase member H-A (BmLHA) was identified as being involved in the response to BmNPV infection in *B. mori* larvae digestive juice. In the present study, a gene encoding the BmLHA protein in *B. mori* was characterized. The protein has an open reading fragment of 999 bp, encoding a predicted 332 amino acid residue-protein with a molecular weight of approximately 35.9 kDa. The phylogenetic analysis revealed that BmLHA shares a close genetic distance with *Papilio xuthus* Lipase member H-A. *BmLHA* was highly expressed in the middle part of the *B. mori* gut, and the expression level increased with instar rising in larvae. There was higher expression of BmLHA in A35 than in P50 strains, and it was upregulated in both A35 and P50 strains, following BmNPV infection. The expression level of *VP39* decreased significantly in appropriate recombinant-BmLHA-treated groups compared with the PBS-treated group in *B. mori* larvae and BmN cells. Meanwhile, overexpression of BmLHA significantly reduced the infectivity of BmNPV in BmN cells. These results indicated that BmLHA did not have digestive function but had anti-BmNPV activity. Taken together, our work provides valuable data for the clarification of the molecular characterization BmLHA and supplements research on proteins of anti-BmNPV activity in *B. mori*.

## 1. Introduction

The silkworm *Bombyx mori L*. (Lepidoptera: Bombycidae) has been domesticated for more than 5000 years and still plays an important role in many developing countries. Moreover, *B. mori* is a good model organism for the study of insect genetics and immunology [1,2]. Infection with viruses, including *B. mori* nucleopolyhedrovirus (BmNPV), is a major cause of silkworm death and leads to the largest sericulture industry losses annually. BmNPV is a major viral pathogen and still remains a significant challenge to the sericulture industry due to a lack of effective prevention methods. BmNPV belongs to the Baculoviridae family and is characterized by a rod-shaped, enveloped virion containing a closed, circular, double-stranded DNA genome of ~130 kilobase-pairs (kb) in length [3,4]. Interestingly, certain *B. mori* strains exhibit high resistance to BmNPV infection [5]. However, the molecular mechanism has not yet been fully elucidated.

In recent years, many genes or proteins related to BmNPV infection response were identified based on utilizing RNA-Seq [6], 2-DE combined MS [7], Label-free [8] and iTRAQ [9] methods. Additionally, some proteins were confirmed to have functions related to BmNPV resistance or infection in *B. mori*. Peptidoglycan recognition protein 2 (BmPGRP2-2) knockdown reduced BmNPV multiplication and mortality in cell lines and in *B. mori* larvae [10]. BmAtlastin-n [11] and *B. mori* C-lysozyme (BmC-LZM) [12] were found to enhance resistance to BmNPV when overexpressed in larvae and cells. Additionally, the *B. mori* Ser/Thr protein phosphatase 2A (BmPP2A) [13] was demonstrated to have an anti-BmNPV function, and Autophagy-related (ATG) protein ATG13 [14], a GP64-binding protein E3 ubiquitin-protein ligase SINA-like 10 (SINAL10) [15] and chaperonin containing t-complex polypeptide 1 (TCP-1) [16], stimulates BmNPV proliferation in BmN cells.

Previous studies have indicated that the enzymes identified from digestive juice not only function to digest food, but also play an important role in weakening or killing pathogens [17,18]. In *B. mori*, some digestive enzymes involved in antiviral activity have already been characterized and verified, such as red fluorescent protein [19], Lipase-1 [20] and serine protease (SP-2) [21]. Alkaline trypsin [22] was also purified from the digestive juice of *B. mori* larvae and showed strong antiviral activity.

Lipases are ubiquitous enzymes in nature, widely distributed in plants, animals and microorganisms. They play a crucial role in fat metabolism by catalyzing the hydrolysis of triacylglycerol to free fatty acids and glycerol [23,24]. In insects, the lipase and lipase homages are related to survivability, reproductive capacity [25], oocyte maturation and development [26], and sex pheromone biosynthesis [27]. Additionally, the lipase-related protein mRNA in midgut showed different expression patterns after challenge with different microorganisms in the Chinese oak silkworm, *Antheraea pernyi* [28]. The intestinal bacterium *Bacillus pumilus* lipase was expressed by prokaryotic expression system, and the antiviral test showed the recombinant lipase could reduce BmNPV infectivity in vitro [29]. The Bmlipase-1 was determined to have strong antiviral activity against BmNPV under in vitro conditions from the digestive juice, and overexpressing Bmlipase-1 could decreased the mortality in *B. mori*, following BmNPV infection [20,30]. Chen et al. [31] found that the relative expression level of *Bmlipase-1* gene was related to the resistance of *B. mori* strains.

In our previous study, based on the label-free proteomics data of *B. mori* larvae digestive juice, we found that the Lipase member H-A(BmLHA) showed upregulation in midgut digestive juice of the resistant strain (A35) compared to the susceptible strain (P50) [32]. There are no relevant reports available in the literature concerning BmLHA in the *B. mori*. To better understand the function of BmLHA and its ability to inhibit BmNPV, the expression profiles of *BmLHA* at different developmental stages, in various tissues and from different resistant strains following BmNPV infection were analyzed. To further define the role of BmLHA during BmNPV infection, the alteration of BmNPV infection in BmN cells were analyzed, following overexpression of BmLHA, using the insect pIZT/V5-His-mCherry vector. Moreover, the effects of virus infection were analyzed after BmNPV was treated with recombinant BmLHA protein in *B. mori* larvae and BmN cells. This study will hopefully promote further investigation into the function of midgut digestive enzymes in response to BmNPV infection and the resistance mechanism of highly resistant *B. mori* strains.

## 2. Materials and Methods

### 2.1. B. mori Rearing and Virus Preparation

The BmNPV-susceptible *B. mori* strain P50 (LC50 = 1.03 × 10^5^ OBs/mL), BmNPV-resistant *B. mori* strain A35 (LC50 = 5.90 × 10^7^ OBs/mL) and BmNPV (T3 strain) were maintained in the Key Laboratory of Sericulture, Anhui Agricultural University, Hefei, China [6]. Each treatment group had 30 *B. mori* larvae, divided into three sample groups (three biological replicates), with ten *B. mori* larvae in each sample. The different tissues were dissected from *B. mori* larvae and washed three times with PBS or DEPC to remove other tissues. Samples were flash-frozen in liquid nitrogen and pulverized. All the samples were stored at −80 °C for later use. The budded virus containing EGFP-tagged (BV-EGFP) was kindly provided by Xue-Yang Wang in the School of Biotechnology, Jiangsu University of Science and Technology, Zhenjiang, China. The number of BV-EGFP (OBs/mL) was confirmed by the end-point dilution assay method.

### 2.2. BmN Cell Culture and Transfection

The *B. mori* ovarian cell line, BmN, was cultured in TC-100 medium supplemented with 10% (*v*/*v*) FBS, 200 µg/mL of penicillin and 100 µg/mL of streptomycin at 28 °C. Transfection was performed by using NeofectTM DNA transfection reagent (NEOFECT, Beijing, China), according to the manufacturer’s instructions. In brief, the cells were seeded in a six-well plate one day in advance, preferably at a cell density of 60%–80% during transfection. Two hours before transfection, we removed the original medium from the cells and replaced it with fresh complete medium. The 2 µg of plasmid DNA with 100 µL of serum-free dilution were mixed thoroughly, to make a DNA dilution. We added 2 μL of Neofect ™ to the DNA dilution solution, at room temperature, for 30 min. Then we added the transfection complex to the cell culture medium and mixed gently. Cellular fluorescence images were used with a Leica inverted research grade microscope DMi3000B camera and processed with the Leica Application Suite V4.6 software.

### 2.3. Bioinformatics Analysis

The cDNA and deduced protein sequence of *Bombyx mori* Lipase member H-A (BmLHA) were analyzed by using DNAMAN 8.0 (Lynnon Corporation, Quebec, QC, Canada). The conserved motif was predicted on the SMART server (http://smart.embl-heidelberg.de/). The signal peptide was predicted by SignalP 4.1 (http://www.cbs.dtu.dk/services/SignalP/). Sequences of orthologs were analyzed by using the BLASTP tool (http://www.ncbi.nlm.nih.gov/). The amino acid sequences of different species were aligned, using the MUSCLE module of the MEGA6 software. A neighbor-joining tree was generated with a bootstrap of 1000 replications, using MEGA6.

### 2.4. RNA Isolation and cDNA Synthesis

The midgut total RNA was extracted, using TRIzol Reagent (Invitrogen, Carlsbad, CA, USA), according to the manufacturer’s instructions. The ratios of A260/280 and the concentrations of the total RNA were determined by a NanoDrop 2000 spectrophotometer (Thermo Fisher Scientific, New York, NY, USA). The integrity was confirmed by 1% agarose gel electrophoresis. The first strand cDNA was synthesized, using PrimeScript RT reagent kit with gDNA Eraser, according to the manufacturer’s instructions (TaKaRa, Osaka, Japan). The internal control primers of *B. mori glyceraldehyde-3-phosphate dehydrogenase* (*BmGAPDH*) were used to evaluate the quality of the cDNA. The qualified cDNA was stored at −20 °C for later use.

### 2.5. Quantitative Reverse Transcription PCR (RT-qPCR)

To analyze the function of selected proteins, the relative expression level of genes was determined by RT-qPCR. Primers used in the RT-qPCR are shown in Table 1. RT-qPCR reactions were prepared with a SYBR Premix Ex TaqTM Kit (TaKaRa, Osaka, Japan), according to the manufacturer’s instructions. In brief, The RT-qPCR reaction was carried out in a 25 μL reaction mixture containing 12.5 μL of SYBR Premix Ex Taq, 9.5 μL of ddH_2_O, 1 μL of forward primer, 1 μL of reverse primer and 1 μL of cDNA template. The reactions were carried out in the CFX96TM Real-Time System (Bio-Rad, Hercules, CA, USA). The thermal-cycling profile consisted of an initial denaturation at 95 °C for 30 s and 40 cycles at 95 °C for 5 s and 60 °C for 30 s. Three biological sample were analyzed, and each sample was performed in triplicate. Relative expression levels were calculated, using the 2^−^^△△Ct^ method [33]. In this study, *BmGAPDH* was selected as an internal control to adjust the data. The statistical analysis was conducted by using ANOVA and an LSD a posteriori test, using SPSS (*p* < 0.05) (IBM, Chicago, IL, USA).

### 2.6. Prokaryotic Expression, Antibody Preparation

The primers (forward 5’-CCCAAGCTTGTAGCTATCCCTACGATTCCC-3’ and reverse 5’-CCGCTCGAGTTAGAAAGGCCAGGCGTT-3’, where the underlined portions indicate the *Hind* III and *Xho* I restriction sites, respectively) were used to amplify fragments. The cDNA of P50 midgut was used as a template. The target fragment of BmLHA was amplified by polymerase chain reaction (PCR) and cloned into the pMD19-T vector. The target fragment ligated into the expression vector pET-32a with restriction enzyme sites *Hind* III and *Xho* I and transformed into *E. coli* (BL21). The resulting proteins were purified, using nickel-nitrilotriacetic acid (Ni-NTA) agarose resin (Qiagen, Hilden, Germany), following the manufacturer’s protocol, and injected into New Zealand White rabbits. Rabbit sera were collected and used as the primary antibody.

### 2.7. Western Blot Analyses

The digestive juice proteins were extracted according to the previous report [32]. The total midguts (without luminal contents and peritrophic membrane) proteins were extracted as previously described [31]. In brief, 1 mL of protein lysis buffer (4% CHAPS, 2 M thiourea, 7 M urea), 0.10 g samples of midgut, 1 mM of phenyl methane sulfonyl fluoride (PMSF) and 10 mg of dithiothreitol (DTT) were added into glass homogenizers for homogenizing. After centrifugation at 12,000× *g* at 4 °C for 40 min, protein concentrations were determined by BCA. The protein extracts (30 μg) were separated on 12% SDS-PAGE gel and transferred to a polyvinylidene fluoride (PVDF) membrane. The PVDF membranes were blocked with 5% non-fat milk in PBST (137 mM NaCl, 2.7 mM KCl, 10 mM Na_2_HPO_4_, 2 mM K_2_HPO_4_, pH 7.5, 0.1% (*v*/*v* Tween-20)) for 2 h at room temperature, followed by three PBST rinses, and finally incubated with primary antibody (1:1000 dilution) for 2 h at room temperature. After washing, antigen–antibody (1:10,000 dilution) in blocking buffer was applied for 1 h. After another series of washes, immobilized conjugates on the membrane were visualized in HRP substrate solution (Tiangen, Beijing, China). Three biologically independent sample were used. The densitometric intensity of protein bands was measured, using ImageJ software (Bio-Rad, Hercules, CA, USA).

### 2.8. Analysis of Viral Propagation in B. mori Midgut Infected by BmNPV with BmLHA Treated

The recombinant-BmLHA was refolded by serial dialyses in 6, 4 and 2 M urea and finally in PBS (137 mM NaCl, 2.7 mM KCl, 10 mM phosphate buffer, 2 mM potassium phosphate, 10% glycerol). The activity of BmLHA was determined by using an ELISA kit for Insect Lipase (Shanghai Enzyme-linked Biotechnology, Shanghai, China), according to the manufacturer’s instructions. The refolded BmLHA was incubated with BmNPV, which was subsequently used to infect *B. mori*, and the level of viral DNA in virus-infected *B. mori* was determined by RT-qPCR. The 100 μL (1 × 10^7^ OBs/mL) of BmNPV was incubated with 900 μL of different concentrations of purified BmLHA for 1 h at room temperature. Meanwhile, 100 μL of the same viral titer of BmNPV was incubated with 900 μL of PBS for 1 h as a control. Next, 5 μL of BmNPV was orally administered to 5th instar larvae. *B. mori* were orally administered with the same virus treated with PBS (control group). Each treatment group had 30 *B. mori* larvae and was divided into three sample groups (three biological replicates), with ten larvae in each sample. After cultivation for 48 h with fresh mulberry, *B. mori* larvae were dissected to collect midgut samples. The genome DNA of midgut was extracted according to the method reported by Maxim et al. [34]. The primer pair *VP39*-F and *VP39*-R were used to amplify the BmNPV *VP39* gene from the extracted DNA by RT-qPCR. The amplified DNA fragments were used to indicate the abundance of viral DNA in virus-infected *B. mori*. Three biologically independent samples were used.

### 2.9. Analysis of Viral Propagation in BmN Cells Infected by BmNPV with BmLHA Treated

Antiviral activity was measured in triplicate, according to the procedure of Selot’s report, with some modification [35]. The refolded BmLHA was incubated with BmNPV, which was subsequently used to infect BmN cells. The level of viral DNA in virus-infected cells was determined by RT-qPCR. The 100 μL (1 × 10^7^ OBs/mL) of BmNPV(BV-EGFP) was incubated with 900 μL of different concentrations of purified BmLHA for 1 h at room temperature. Meanwhile, an equivalent volume of PBS was used instead of the recombinant-BmLHA in the negative control group. Then, the homogenate was centrifuged at 15,000× *g* for 30 min at 4 °C, and the supernatants were discarded. Then, 1 mL of TC-100 cell-medium was added to the tube for the next step. The mixture was further centrifuged at 15,000× *g* for 30 min at 4 °C, to discard the supernatants, and 1 mL of TC-100 cell culture-medium was added to tube, mixed well and swirled for 10 min. The mixture was sterilized by filtration, using a 0.22 μm pore membrane (Millipore, Boston, MA, USA). BmN cells were seeded in six-well culture plates and allowed to attach at 28 °C. The cells in each well were infected BV with PBS-pretreated or recombinant-BmLHA-treated, respectively. Then, the BVs in the supernatant was removed by replacing the medium with fresh medium after 12 h of incubation. The cells were collected at 28 °C for the 24 h post-infection (hpi), 48 hpi and 72 hpi timepoints. The genomic DNA of BmN cells was extracted according to the method reported by Maxim et al. [34]. The primer pair *VP39*-F and *VP39*-R was used to amplify the BmNPV *VP39* gene from the extracted DNA by RT-qPCR. The amplified DNA fragments were used to indicate the abundance of viral DNA in virus-infected BmN cells. Three biologically independent sample were used.

### 2.10. Overexpression of BmLHA in BmN Cells and BmNPV Infection

The functional domain of BmLHA was amplified with BmLHA primers: F-5’-CCCAAGCTTATGAAACTCTTCATAGCGCTTGC-3’ and R-5’-CCGCTCGAGGAAAGGCCAGGCGTTGCG-3’, where the underlined portions indicate the *Hind* III and *Xho* I restriction sites, respectively). Purified PCR products were ligated into pMD-19T for sequencing. The functional domain of BmLHA was obtained by digestion from the recombinant plasmid, using *Hind* III and *Xho* I, and subsequently ligated into the pIZT/V5-His-mCherry plasmid, to construct the transient expression vector pIZT/V5-His-mCherry-BmLHA. BmN cells (1 × 10^6^ cells/well) were seeded on to Costar 6-well cell culture clusters and cultured overnight at 28 °C. Transfection was performed using NeofectTM DNA transfection reagent (NEOFECT, Beijing, China). Each transfection was repeated three times. After a 48 h incubation with the overexpression vector, BmN cells were harvested to extract the RNA and protein. The overexpression efficiency of BmLHA was assayed by RT-qPCR. The BmN cells in each well were infected with BV-EGFP, respectively. The BmN cells were collected at 28 °C for 24 hpi, 48 hpi and 72 hpi timepoints, and genomic DNA of BmN cells was extracted according to the method reported by Maxim et al. [34]. The primer pair VP39-F and VP39-R was used to amplify the BmNPV VP39 gene from the extracted DNA by RT-qPCR. The amplified DNA fragments were used to indicate the abundance of viral DNA in virus-infected BmN cells. Three biologically independent samples were used.

### 2.11. Cell Viability Counting Assay

A Cell Counting Kit-8 (CCK-8) (Dojindo, Shanghai, China) was used to measure cell proliferation and detect cytotoxicity, according to the manufacturer’s instructions. Briefly, BmN cells were transfected with pIZT/V5-His-mCherry or pIZT/V5-His-mCherry-BmLHA. Cell were collected in 100 μL aliquots at 24, 48 and 72 h. These 100 μL aliquots were added to individual wells in 96-well plates, together with 10 μL of CCK-8, and incubated for 2 h. Absorbance was measured with a spectrophotometer, at a wavelength of 450 nm. There were three biological sample replicates, and each biological sample replicate included three technique replicates.

## 3. Results

### 3.1. Analysis and Validation of BmLHA Omics Data

**Figure 1:**
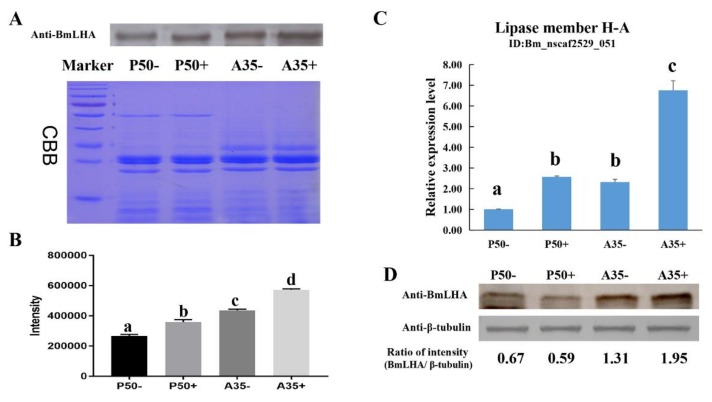
The expression levels of BmLHA in digestive juice and midgut of the different strains, following BmNPV infection. (**A**) The protein levels of BmLHA in P50−, P50+, A35− and A35+. Thirty micrograms of midgut digestive juice protein was analyzed by Western blot with BmLHA antiserum (top). Total proteins were Coomassie Brilliant Blue (CBB)-stained as the loading control (bottom); (**B**) densitometric intensity analysis of the Western blot by using Image J software; (**C**) the BmLHA expression levels in midgut of P50−, P50+, A35− and A35+, at 24 h post-inoculation. Relative expression levels were calculated by using the 2^−^^∆∆Ct^ method; (**D**) the protein levels of BmLHA in P50−, P50+, A35− and A35+ and densitometric intensity analysis of the Western blot by using Image J software. Thirty micrograms each of midgut protein was analyzed by Western blot with antiserum (top). The β-tubulin as the loading control (bottom). “−”; non-treated, “+”; BmNPV-infection. Statistical analysis was conducted by using the SPSS software. Significant differences are indicated by different letters (*p* < 0.05).

In our previous study, a quantitative label-free proteomic analysis revealed differentially expressed proteins (DEPs) in the digestive juice of resistant (A35) versus susceptible (P50) *B. mori* strains. Based on the label-free database and NCBI BlastP, we obtained the seven unique peptides of Lipase member H-A(BmLHA) (GenBank Accession No: XP_004932346.1) (Table 2). In previous research [32], DEPs were defined based on a 1.5-fold change threshold (with a fold change > 1.5 or < 0.67, *p* < 0.05). The expression level of BmLHA in A35+ was increased three-fold (BmNPV-infection) compared to A35− (non-treated), while no significant differences occurred in P50+ (BmNPV-infection) compared to P50− (non-treated). The expression level of BmLHA was increased three-fold in A35− compared to P50−, and the expression level of BmLHA was increased nine-fold in A35+ compared P50+ (Table 2). Western blotting was used to further confirm the observed changes in protein expression in the A35 and P50 strains, following BmNPV infection. Because no appropriate control for digestive juice exists, Coomassie Blue R-250 staining was performed according to the method of Rao et al. [36]. The results demonstrated that the expression level of BmLHA was increased in A35+ compared to A35−, and the same trend occurred in P50+ compared to P50−. The Western blot results demonstrated that the protein levels of BmLHA were consistent with the trends observed with the label-free data (Figure 1A,B). Because the midgut juice was secreted by midgut cells, the expression level of transcription and protein of BmLHA in the midgut were analyzed by RT-qPCR and Western blot. The RT-qPCR results show that the expression levels of *BmLHA* were increased in the A35 and P50 strains, following BmNPV infection (Figure 1B), and the Western blot results show that the expression levels of BmLHA were consistent with the label-free data in digestive juice (Figure 1C).

### 3.2. Characterization of the BmLHA Sequence

**Figure 2:**
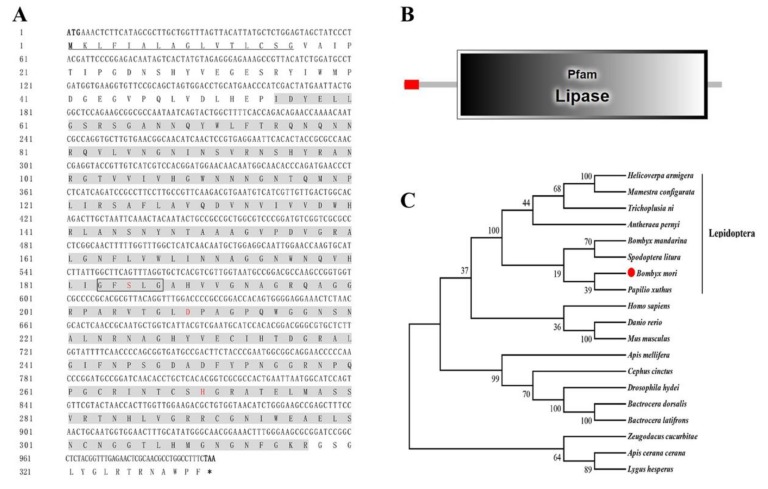
Bioinformatics analysis of BmLHA. (**A**) The ORF nucleotide sequence of BmLHA and its deduced amino acid sequence. The ORF of BmLHA is 999 bp encoding a 332 amino acid protein with a molecular weight of 35.93 kDa. The signal peptide (1–16) was predicted by SignalP 4.1. The Lipase domain is shaded gray. Consensus sequence (GXSXG) in box and residues of the catalytic site are labeled in red. The cDNA sequence is deposited in GenBank under accession number XM_004932289.1; (**B**) gene structure analysis of BmLHA by using SMART online software; (**C**) phylogenetic relationships of BmLHA in different species, using the neighbor-joining method, with a bootstrap value of 1000.

To further investigate the function of BmLHA, a bioinformatics analysis was performed. The cDNA (GenBank Accession No: XM_004932289) sequence of BmLHA contains an ORF of 999 bp, which encodes 332 amino acids with a predicted size of 35.93 kDa and an isoelectric point of 9.17. Additionally, the first 16 amino acid residues at the N-terminus of BmLHA contained a sequence (1–16) that acts as a signal peptide for secretion (Figure 2A) and indicated that BmLHA is a secreted protein. The BmLHA conserved domain prediction, using SMART software, indicated that the BmLHA protein contains a Lipase domain (56–317) (Figure 2B), suggesting that BmLHA belongs to the Lipase family. To investigate the evolutionary relationships between BmLHA and those of other insects, a phylogenetic tree was constructed by the neighbor-joining method (Figure 2C). The results showed that BmLHA and its homologs from seven other insects were clustered into the Lepidoptera group (Figure 2C), and it shares a close genetic distance with *Papilio xuthus*. Amino acid sequence multiple alignment analysis revealed that BmLHA shared 16%–26% identity with the lipase member H-A from other insect species (Figure S1).

### 3.3. The Analyzed of Expression Pattern of BmLHA

**Figure 3:**
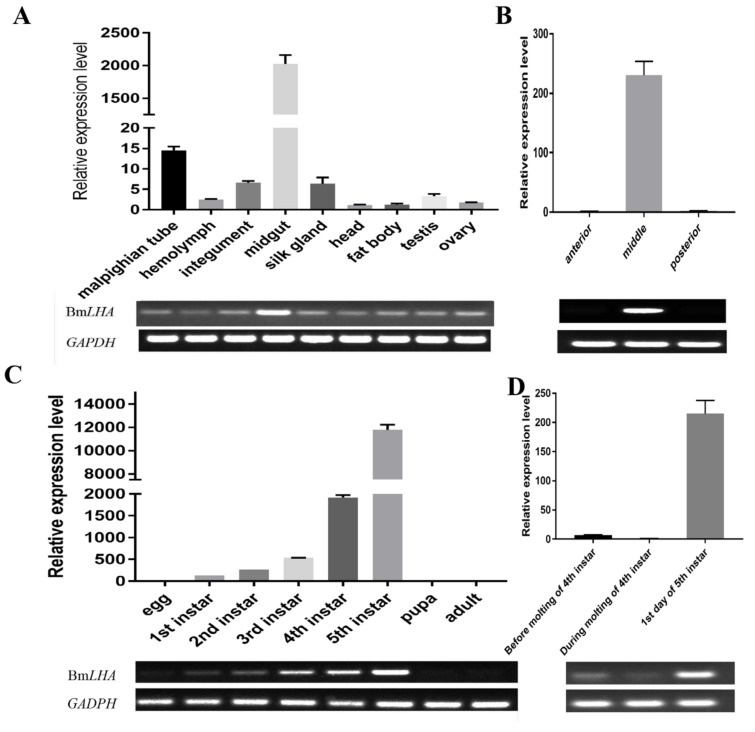
Expression profiles of *BmLHA* in *B. mori*. (**A**) RT-qPCR and RT-PCR analysis of the expression level of *BmLHA* in different tissues; (**B**) RT-qPCR and RT-PCR analysis of the expression level of *BmLHA* in *B. mori* foregut, midgut and hindgut; (**C**) RT-qPCR and RT-PCR analysis of the expression level of *BmLHA* at different developmental stages; (**D**) RT-qPCR and RT-PCR analysis of the expression level of *BmLHA* during, before and after moulting. The *BmGADPH* was used as an internal control. Triple experiments were performed to calculate the values for relative levels of *BmLHA* transcripts.

In order to analyze the transcriptome patterns of *BmLHA* in different tissues and at various developmental stages, the total RNA was extracted, and the RT-qPCR and RT-PCR analyses were performed. The results showed that the *BmLHA* was expressed at the highest concentration in the *B. mori* midgut, and expressed at lower levels in the Malpighian tube; expression was very low in other examined tissues (Figure 3A). The *BmLHA* was expressed in the middle of the *B. mori* gut, and there was no expression in the foregut and hindgut (Figure 3B). Additionally, the expression level of *BmLHA* significantly increased form the first instar to the fifth instar, while no expression was observed in egg, pupa or adult (Figure 3C). The *BmLHA* demonstrated higher expression on the first day of the fifth instar, compared to the fourth day of fourth instar before moulting stage, while a very low expression was observed during the moulting stage (Figure 3D).

### 3.4. Variation of BmLHA Expression Level Following BmNPV Infection in Different Resistant Strains

**Figure 4:**
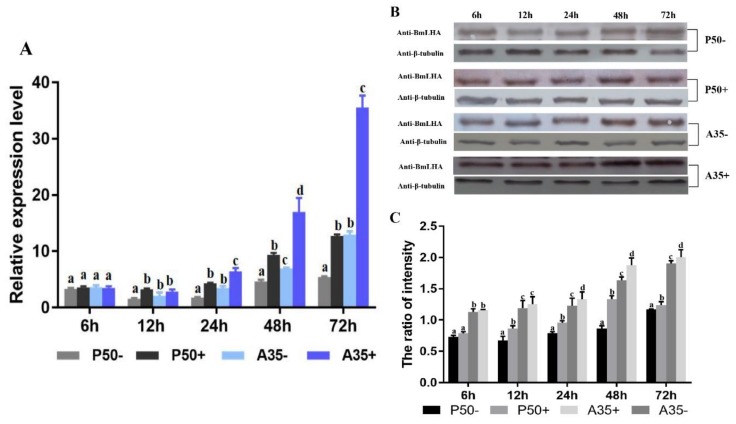
Relative expression level of BmLHA in the midgut after BmNPV infection in different strains of *B. mori*. (**A**) The BmLHA transcription expression levels in midgut of P50−, P50+, A35− and A35+ at 6, 12, 24, 48 and 72 h. Relative expression levels were calculated by using the 2^−^^∆∆Ct^ method; (**B**) the BmLHA protein expression levels in midgut of P50−, P50+, A35− and A35+ at 6, 12, 24, 48 and 72 h; (**C**) densitometric intensity analysis of the Western blot by using Image J software. Statistical analysis was conducted by using the SPSS software. Significant differences are indicated by different letters (*p* < 0.05).

To investigate the variation of BmLHA in the responses to BmNPV infection, RT-qPCR and Western blotting were conducted to measure BmLHA transcript and protein abundance in different strains of *B. mori* midgut. The RT-qPCR results indicated that *BmLHA* expression levels were upregulated in P50+ compared to P50− from 12 to 72 h after *BmNPV* infection and increased along with the time (Figure 4A). Additionally, *BmLHA* transcripts showed upregulated in A35+_vs._A35− from 24 to 72 h after BmNPV infection, and increased along with the time (Figure 4A). Surprisingly, the expression level of *BmLHA* was upregulated in A35−, compared to P50− from 12 to 72 h. Levels were also increased in A35+ versus P50+ strains from 24 to 72 h (Figure 4A). The Western blot results demonstrated that the protein levels of BmLHA were consistent with the trends observed with the transcript levels in different resistant strains, following BmNPV infection. Meanwhile, the expression level of BmLHA in the A35 strain was higher than that of the P50 strain from 6 to 72 h (Figure 4B,C). These results indicated that BmLHA could respond to BmNPV infection in *B. mori* midgut.

### 3.5. Antiviral Effects Analysis of BmLHA in B. mori Larvae

**Figure 5:**
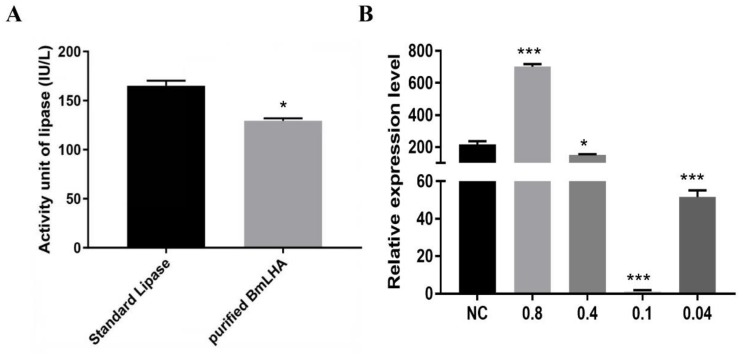
Antiviral effects of recombination-BmLHA in *B. mori* larvae. (**A**) Assay of recombination-BmLHA lipase activity the vitro; (**B**) the effect of BmNPV infection with different concentrations of recombination-BmLHA. The relative expression level of BmNPV *VP39* gene in *B. mori* midguts was detected by RT-qPCR at 48 h after BmLHA treatment during BmNPV oral infection. Statistical analysis was conducted by using the SPSS software. The difference between the samples was analyzed by the *t*-test method. Significant differences are indicated by asterisks (*, *p* < 0.05; **, *p* < 0.01; ***, *p* < 0.001). NC, PBS-treated.

We sought to investigate the antiviral effect of BmLHA in *B. mori* against BmNPV infection. The recombinant-BmLHA was expressed, using a prokaryotic expression system (Figure S2), and refolded via refolding buffer. The lipase activity of recombinant BmLHA was subsequently assayed. The lipase activity of recombinant BmLHA was 130 IU/L, compared with 180 IU/L for the lipase standard supplied with the assay kit, and recombinant BmLHA could be used to subsequent experiments (Figure 5A). To further validate the function of BmLHA related to BmNPV infection, the change of viral copy number was used to measure the effect of BmLHA on BmNPV infectivity. The proliferation analysis of BmNPV in the presence of different concentrations of recombinant-BmLHA-treated groups at 48 hpi was detected by using RT-qPCR. The expression level of *VP39* was downregulated in the groups of 0.4, 0.1 and 0.04 mg/mL recombinant-BmLHA-treated compared to PBS-treated groups (Figure 5B). The expression level of VP39 was significantly downregulated in BmNPV with 0.1 mg/mL compared to 0.4 and 0.04 mg/mL recombinant-BmLHA (Figure 5B). Interestingly, the expression level of *VP39* was upregulated in the group with 0.8 mg/mL recombinant-BmLHA compared to PBS-treated group. This result showed that the appropriate concentration recombinant-BmLHA could inhibit the BmNPV infectivity, but a higher concentration of recombinant-BmLHA could affect the *B. mori* larva’s ability to resist the BmNPV infection.

### 3.6. Antiviral Effects Analysis of BmLHA in BmN Cells

**Figure 6:**
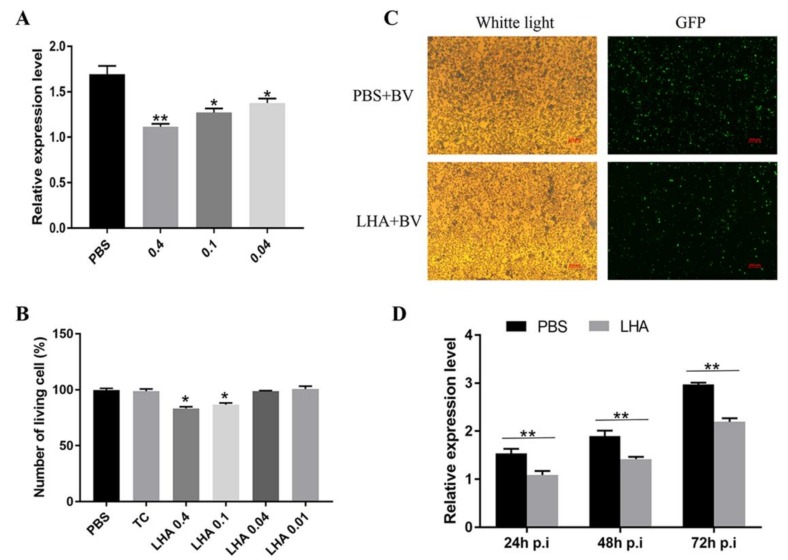
Antiviral effects of recombination-BmLHA on BmN cells. (**A**) Proliferation of BmNPV was assessed using RT-qPCR by analyzing relative expression levels of *VP39* at 48 hpi; (**B**) cell proliferation and cytotoxicity assays were used to detect the cell viability of these treated cells; (**C**) the infected cells (EGFP positive) were examined, using fluorescence microscopy, at 72 hpi. Scale bar = 250 µm. Trans (white), optical transmission. EGFP (Green), expressed wing the replication of BVs; (**D**) proliferation of BmNPV was assessed, using RT-qPCR, by analyzing relative transcription levels of *VP39* at 24, 48 and 72 hpi. Statistical analysis was conducted by using the SPSS software. Significant differences are indicated by asterisks (*, *p* < 0.05; **, *p* < 0.01).

The above results showed the effect of the recombinant-BmLHA on ODV infectivity. To further study the effect of recombination-BmLHA on BV infectivity, the amount of viral DNA in BmN cells infected with the recombinant-BmLHA-treated and PBS-treated BV was investigated. The relative VP39 gene expression level was analyzed by using RT-qPCR at 48 hpi (Figure 6A). The results showed that the virus proliferation could be inhibited by concentrations of 0.04, 0.1 and 0.4 mg/mL recombinant-BmLHA, and the efficiency of inhibition increased proportionally to recombinant-BmLHA concentration. At the same time, as measured by cell viability counting assay, the cell proliferation was not normal in the groups treated with a concentration of recombinant-BmLHA greater than 0.04 mg/mL, when compared to the control group (Figure 6B). We thus selected the 0.04 mg/mL concentration of recombinant-BmLHA for subsequent experiments. The BmN cells were infected by BV treated with 0.04 mg/mL of recombinant-BmLHA or PBS. At 72 hpi, the GFP fluorescence intensity of PBS-treated groups was greater than those that were treated with recombinant-BmLHA (Figure 6C). Additionally, the expression level of *VP39* was analyzed by RT-qPCR at 24, 48 and 72 hpi. The results showed that the *VP39* expression level of groups treated with recombinant-BmLHA were lower than groups treated with PBS at all time points (Figure 6D). The results showed that the recombinant-BmLHA reduced the infectivity of BVs and the virus DNA replication was partly inhibited in BmN cells.

### 3.7. Antiviral Effects Analysis in BmLHA Overexpressed BmN Cells

**Figure 7:**
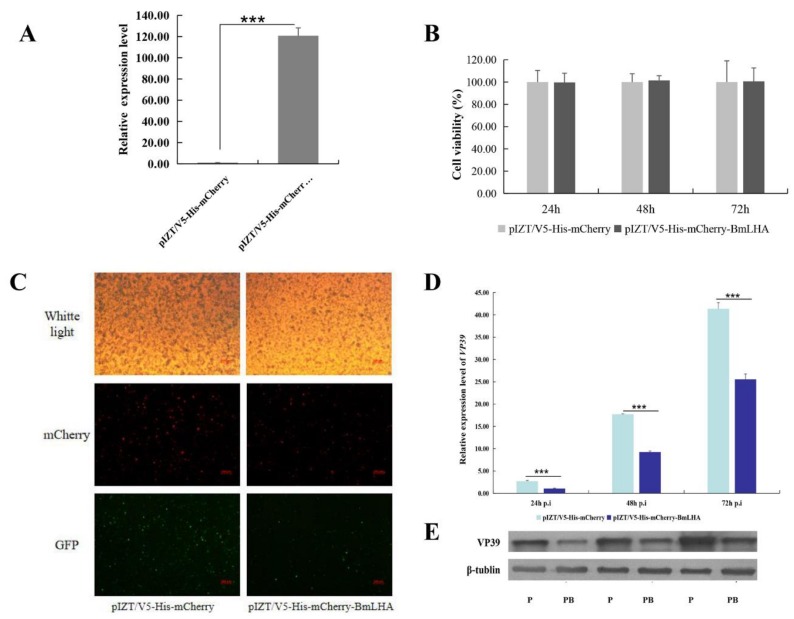
Infection analysis of BmNPV in BmN cells. (**A**) The transcriptional level of BmLHA in BmN cells transfected with pIZT/V5-His-mCherry/BmLHA at 48 h after transfection; (**B**) cell proliferation and cytotoxicity assays were used to detect the cell viability of these transfected cells; (**C**) the infected cells (EGFP positive) were examined by using fluorescence microscopy at 72 hpi. Scale bar = 250 µm. Trans (white), optical transmission. EGFP (Green), expressed wing the replication of BV; mCherry (Red), fused expression with BmLHA protein. pIZT/V5-His-mCherry was negative control; (**D**) proliferation of BmNPV was assessed with RT-qPCR by analyzing relative expression levels of *VP39* at 24, 48 and 72 hpi; (**E**) Western blot of viral protein VP39 expression levels at 24, 48 and 72 hpi. (“P” pIZT/V5-His-mCherry; “PB” pIZT/V5-His-mCherry-BmLHA). The data were normalized by using *BmGAPDH* and are represented as the mean ± standard error of the mean, from three independent experiments. Relative expression levels were calculated by using the 2^−∆∆Ct^ method. Statistical analysis was conducted, using the SPSS software. Significant differences are indicated by asterisks (*, *p* < 0.05; **, *p* < 0.01; ***, *p* < 0.001).

To analyze the effect of BmLHA on BmNPV infectivity in vivo, the insect expression vector pIZT/V5-His-mCherry was used to overexpress BmLHA in BmN cells. The purified PCR product of BmLHA ORF was inserted into the pIZT/V5-His-mCherry vector using *Hind III* and *Xho I* restriction enzymes. BmN cells were transfected with the recombinant bacmid to overexpress BmLHA. The red fluorescence protein of mCherry indicates that BmLHA was successfully overexpressed in BmN cells (Figure 7C), which was validated by RT-qPCR (Figure 7A). Cell proliferation and cytotoxicity assays indicated that overexpression of BmLHA did not affect cell growth (Figure 7B). To further analyze antiviral effects in BmLHA-overexpressed BmN cells, the expression level of VP39 in overexpressed-BmLHA BmN cells and controls at different infection times was analyzed by RT-qPCR and Western blot. These results indicate that the BV replication in overexpressed-BmLHA cells was significantly lower than that of in the control at all three time points (Figure 7D,E). This suggests that BmLHA could inhibit the BmNPV infection in BmN cells.

## 4. Discussion

Lipases (triacylglycerol acylhydrolases, EC 3.1.1.3) are water-soluble enzymes that act on non-soluble substrates. Lipases play an important role in lipid metabolism and energy homeostasis because fatty acids, mostly stored as triacylglycerides (TAG), are the major endogenous source of energy [37]. Insect lipases have key roles in lipid acquisition, storage and mobilization, and they are also fundamental to many physiological processes, such as insect reproduction, development, defense against pathogens, oxidative stress and pheromone signaling [25,26,27,28,30]. Additionally, the lipase-like gene (ORF19) from Heliothis virescens ascovirus (HvAV-3e) has been shown to be essential for virus replication and cell cleavage [38]. The lipase family proteins not only play a crucial role in fat metabolism but are also involved in viral infection. In our previous comparative proteomics analysis of different resistant *B. mori* midgut digestive juice in response to BmNPV infection [32], BmLHA showed significant changes in expression in the resistant strain (A35) following BmNPV infection, while no significant differences were observed in the susceptible strain (P50). We suspected that BmLHA had antiviral activity in *B. mori* larvae digestive juice.

In the present study, we identified a BmLHA from *B. mori* midgut juice via the label-free database. We have named it BmLHA and enrolled it in GenBank, Accession No: XP_004932346.1. The domain analysis showed that BmLHA contains a signal peptide at the N-terminus and the lipase putative catalytic triad consensus sequence (GXSXG), suggesting that it is a secreted protein and belongs to the lipase family. The phylogenetic analysis revealed that BmLHA shares a close genetic distance with *Papilio xuthus*. These results will provide a foundation for further analyses into the biological function of BmLHA.

The tissue expression profiles of BmLHA indicated that it was strongly expressed in the midgut. The midgut is an import tissue for food digestion [18]. Many digestive-related proteases are only expressed in the midgut or are highly expressed in the midgut. For example, the serine proteases (SP), a major class of digestive proteases accounting for 95% of digestive activity in Lepidoptera, showed similar expression profiles to that of BmLHA in the *B. mori* [39,40,41,42]. While the *BmLHA* expressed at lower levels in the Malpighian tube and other tissues. The lipase-related protein tissue expression profiles showed similar trend in the Chinese oak silkworm, *Antheraea pernyi* [28]. The results suggested that the *BmLHA* had multiple function in *B. mori. BmLHA* was specifically expressed in the middle of the *B. mori* gut, and there was no expression in the foregut and hindgut. We predicted that BmLHA is mainly synthesized in midgut cells and released into gut lumen for performance of essential digestive functions. Furthermore, the expression level of *BmLHA* was higher in the first day of fifth instar compared to the fourth day of fourth instar prior to the moulting stage, and demonstrated very low expression during the moulting stage. Liu et al. [40] found that the expression level of *BmSP36* was regulated by juvenile hormone (JH), with the same expression profiles in different moulting stages. At the same time, Zhang et al. [27] demonstrated that *B. mori pancreatic lipase-like gene* (*BmPLLG*) is involved in pheromone biosynthesis via activation of neuropeptide (PBAN)-induced sex pheromone biosynthesis and released by influencing bombykol production. It is also suggested that the expression of *BmLHA* may be regulated by hormones. Additionally, the *BmTCP-1*, *B. mori heavy* and *light chain homolog* (*BmHCH, BmLCH*) and *B. mori Cytochrome c* (*BmCytc*) genes found to respond to BmNPV infection were regulated by hormones [16,43,44].

The BmLHA transcript and protein abundance in different strains of *B. mori* indicated that the expression level of BmLHA was upregulated in A35+ and P50+ compared to A35− and P50−, respectively. A previous study also reported similar results, showing that expression levels of *lipase-1*, which belongs to the Lipase family, were upregulated in three different resistant strains, following BmNPV infection [8]. Meanwhile, the results in the present study indicated that the expression level of BmLHA was higher in A35− than P50−. Chen et al. [31] found that the *lipase-1* expression was significantly higher in the resistant strains compared to susceptible strains. Additionally, we noted the BmLHA was highly expressed in the A35 strain, following BmNPV infection. The antivirus protein alkaline trypsin displayed similar results in different resistant strains, following BmNPV infection [22]. These results confirmed that BmLHA could respond to BmNPV infection and suggested that BmLHA is likely to play an important role in resistance to BmNPV infection in the *B. mori*.

In a previous study, it was observed that the lipase-1 from the digestive juice of *B. mori* larvae demonstrated strong antiviral activity against BmNPV in vitro [20]. Additionally, the lipase from intestinal bacterium *B. mori* Linnaeus SW41 could reduce BmNPV infectivity in vitro [29]. In this study, the antiviral test showed that an appropriate concentration of the recombinant-BmLHA reduced BmNPV infectivity in vitro, resulting in decreased viral DNA abundance in *B. mori* larvae and BmN cells. The protein-viral incubation method also suggested that the recombinant-BmLHA is potentially acting directly on the BmNPV by destroying viral integrity and consequently reducing the infectivity of BmNPV [20,29]. These results indicated that the BmLHA had an antiviral activity similar to other digestive juice proteins, such as Lipase-1 [20], SP-2 [21], Alkaline trypsin [22] and soluble NADPH oxidoreductase (BmNOX) [35]. However, we also observed that the number of viral DNA copies in groups treated with higher concentrations of recombinant-BmLHA were increased compared to the PBS-treated groups. We speculate that the viral infection was accelerated after the physiological balances of the host were disturbed by a higher concentration recombinant-BmLHA, although the recombinant-BmLHA could reduce the infectivity of BmNPV. Cell viability results showed that high concentrations of recombinant-BmLHA could affect host homeostasis. These results suggest that the recombinant-BmLHA had antiviral activity in *B. mori* larvae and BmN cells, but could destroy host homeostasis with the higher concentrations. A detailed analysis of this mechanism needs to be explored in the future.

Some endogenous genes involved in the *B. mori* defense response against BmNPV were confirmed in previous studies. A transgenic *B. mori* with a vector overexpressing the Lipase-1 [30], BmAtlastin-n [11] and BmC-LZM [12] could increase anti-BmNPV capacity of larvae compared with the non-transgenic line challenged with BmNPV. Additionally, The BmN cells with a vector overexpressing the antivirus genes, such as BmSP142 [39], BmPP2A [13] and BmThymosin [45], could reduce the number of viral copies compared with the control line following the BV infection. This evidence demonstrates that overexpressing endogenous antiviral genes can enhance the antiviral resistance of *B. mori* or BmN cells. In this study, BmLHA was overexpressed, using the overexpression insect vector, by inserting the ORF of BmLHA and transfecting in BmN cells. The number of viral copies in the overexpressed BmLHA cells was significantly lower than in the control, following the BV infection. Therefore, we confirmed that BmLHA, as with other antiviral proteins, was involved in resistance to BmNPV infection in BmN cells. These findings demonstrate that BmLHA could inhibit replication of BmNPV in BmN cells.

## 5. Conclusions

In summary, we identified a BmLHA in the *B. mori* midgut digestive juice proteome database. Gene expression analysis revealed that BmLHA was highly expressed in the midgut and may be regulated by hormones. In response to BmNPV infection, the expression level of BmLHA was upregulated in the two *B. mori* strains and highly expressed in A35 strain, following BmNPV. In addition, the appropriate concentration of recombinant-BmLHA could inhibit the BmNPV infectivity in *B. mori* larvae and BmN cells. Meanwhile, overexpression of BmLHA significantly prolonged the infection process of BmNPV in BmN cells. Taken together, this study lays a foundation for studying the function of antiviral infection of digestive juice and exploring the resistance mechanisms in *B. mori*.

## Figures and Tables

**Table 1:** Primers used in RT-qPCR.

Gene ID	Forward Primer (5′-3′)	Reverse Primer (5′-3′)
*BmLHA*	GTGATGCCGACTTCTACCCG	ACCACAGCGTCTTCCAACCA
*VP39*	CAACTTTTTGCGAAACGACTT	GGCTACACCTCCACTTGCTT
*BmGAPDH*	CGATTCAACATTCCAGAGCA	GAACACCATAGCAAGCACGAC

**Table 2:** Differentially expressed protein BmLHA by label-free in digestive juice of different resistant strains, following BmNPV infection.

Protein ID	Protein Description	Unique Peptides	P50+_vs._ P50− Ratio	A35+_vs._ A35− Ratio	A35−_vs._ P50− Ratio	A35+_vs._ P50+ Ratio
Bm_nscaf 2529_051	Lipase member H-A	ALGIFNPSGDADFYPNGGRЖ ATELMASSVRЖ LANSNYNTAAAGVPDVGRЖ NAGHYVECIHTDGRЖ QVLVNGNINSVRЖ SAFLAVQDVNVIVVDWHRЖ VTGLDPAGPQWGGNSNALNR	1.296	3.025	4.164	9.716

## Supplementary Materials

The following are available online at https://www.mdpi.com/2075-4450/11/3/154/s1, Figure S1: Amino acid sequence multiple alignments of BmLHA with homologs from other insect species, Figure S2: Analysis of recombinant BmLHA protein using SDS-PAGE and Western blotting.
