# Supplementary material for: A Novel Digestive Proteinase Lipase Member H-A in Bombyx mori Contributes to Digestive Juice Antiviral Activity against B. mori Nucleopolyhedrovirus
Source: Insects. 2020 Mar 1;11(3):154. doi: 10.3390/insects11030154 (PMC7143000; doi:10.3390/insects11030154)
Supplement: Supplementary file 1 [file insects-11-00154-s001.pdf]

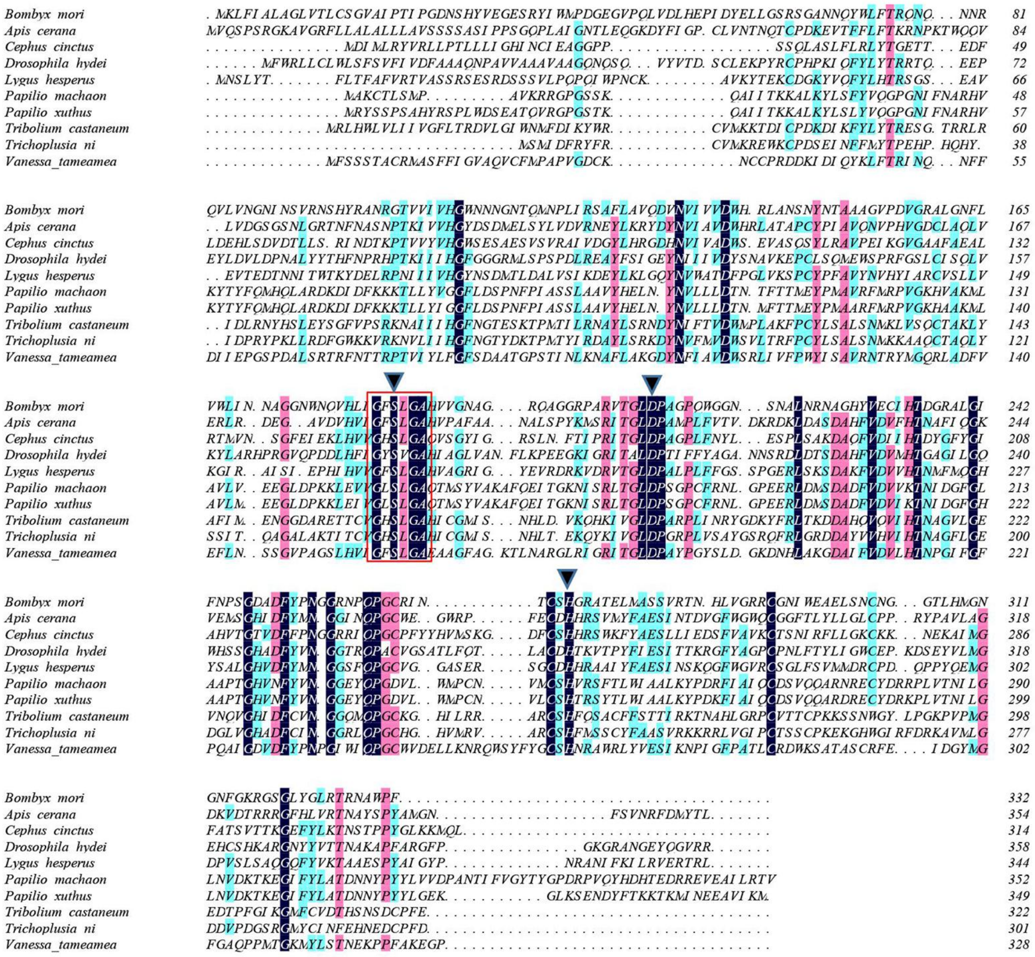

Figure S1. Amino acid sequence multiple alignments of BmLHA with homologs from other insect species. Black represents 100% identity, grey represents 80% identity and white represents < 80% identity. Arrow: indicates location of the putative catalytic triad, box: indicates consensus sequence (GXSXG).

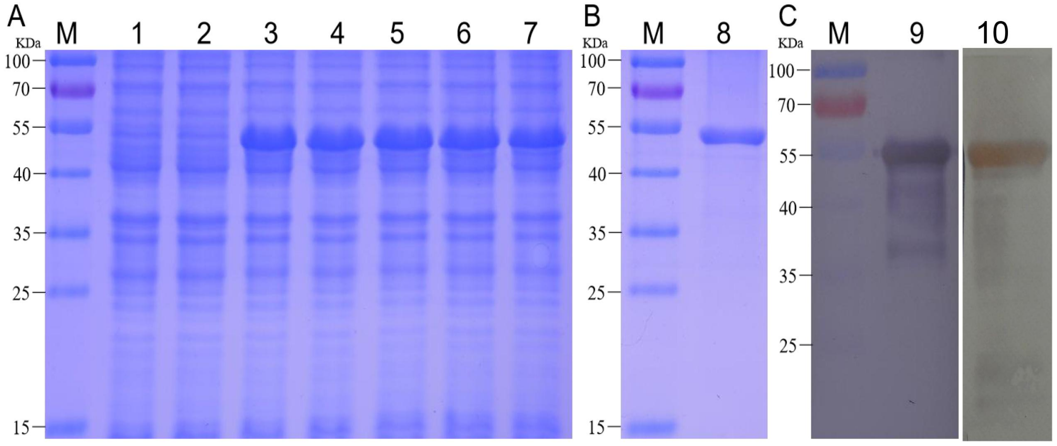

Figure S2. Analysis of recombinant BmLHA protein using SDS-PAGE and Western blotting. Recombinant BmLHA protein was induced by different concentration of IPTG. M: molecular weight markers. Lane 1: blank control without insert. Lane 2: negative control without induction. Lane 3–7: induced expression under IPTG concentrations of 0.2 mM, 0.4 mM, 0.6 mM, 0.8 mM and 1.0 mM. Lane 8: purified recombinant BmLHA protein.

Lane 9 and 10: Western blotting analysis of recombinant His-tagged BmLHA protein identified by anti-His antibodies and anti-BmLHA antibodies, respectively.
